# Non-symbolic magnitudes are represented spatially: Evidence from a non-symbolic SNARC task

**DOI:** 10.1371/journal.pone.0203019

**Published:** 2018-08-30

**Authors:** Fiona Nemeh, Judi Humberstone, Mark J. Yates, Robert A. Reeve

**Affiliations:** 1 Melbourne School of Psychological Sciences, University of Melbourne, Victoria, Australia; 2 Department of Neurological Surgery, Columbia University, New York, New York, United States of America; University of Rome, ITALY

## Abstract

A core proposition in numerical cognition is numbers are represented spatially. Evidence for this proposition comes from the “spatial numerical association of response codes” effect (SNARC) in which faster responses are made by the left/right hand judging whether one of a pair of Arabic digits is smaller/larger than the other. Less is known if a similar SNARC effect exists for non-symbolic magnitudes; and research that has been conducted used stimuli which could be translated into symbolic terms. To overcome this limitation, we employed a referent-to-target judgment paradigm in which a referent dot array (*n* = 30 dots) was follow by a second array of dots (e.g., *n* = 45 or 15 dots)–participants judged if the second array contained fewer or more dots than the referent array. Dot arrays with fewer dots were judged more quickly with the left hand compared to the right hand (i.e., a SNARC effect). Not all participants demonstrated a SNARC effect, however. Neither visuospatial working memory nor math ability was associated with the presence/absence of a non-symbolic SNARC effect. Implications of the non-symbolic SNARC effect for accounts of numerical cognition are discussed.

## Introduction

The brain appears to associate numbers with spatial locations: smaller numbers are associated with the left side of space and larger numbers with the right side. The “spatial-numerical association of response codes” (SNARC) illustrates this association: The speed of magnitude judgement differs as a function of the hand used to make the judgement [1]. It has been suggested the SNARC effect activates a cognitive representation of number and occurs because of the association between the spatial code of the side of response and the magnitude of the number [2–5]. With smaller numbers (e.g., 1 or 2), judgments are faster using the left hand, and with larger numbers (e.g., 8 or 9), judgments are faster using the right hand. The SNARC effect is found for visual number words, auditory number words [6], double-digit numbers [7], and negative numbers [8].

The SNARC effect has also been found in non-numerical domains such as letters of the alphabet [9], object size [10–11], pitch height [12–13], loss and gain words [14], and emotional valence [15] (see Macnamara, Keage & Loetscher, for a review [16]). Given non-symbolic magnitude representation may be a more basic form of quantity representation (evident in human infants and animals), which may support symbolic representation, it is important to determine if a SNARC effect exists for non-symbolic quantity information.

The parietal area of the brain appears to represent number in an abstract notation form (i.e., activated by “3”, “three”, or three objects [17]), which suggests smaller and larger non-symbolic magnitudes (e.g., dot patterns with a smaller or larger numbers of dots) might be associated with spatial locations. Numerosity appears to be mapped topographically in the parietal cortex [18]. The parietal area is also involved in spatial processing [19–22]. Indeed, some findings have been interpreted as showing a spatial-numerical association for non-symbolic quantities using SNARC or SNARC-like paradigms [6, 23–25]. However, caution should be exercised since these findings could be interpreted in other ways.

It is possible that symbolic number representations are activated by non-symbolic stimuli, which implies sub-vocally counting cannot always be discounted. Non-symbolic magnitudes occasionally span the subitizing and approximate number system ranges, which activate different neuro-cognitive systems [26]. The approximate number system—the ANS—allows representations of large approximate non-symbolic magnitudes, whereas the subitizing range focuses on small precise magnitudes (e.g., “1”–“4”) (see Feigenson, Dehaene & Spelke [27]). Evidence for this possibility has been found by Mitchell et al., [23] who showed a SNARC-like effect in a task for which non-symbolic magnitude stimuli were irrelevant to the task at hand. Participants judged the colour of dots (Experiment 1) or the orientation of triangles which were displayed as upright or inverted (Experiment 2). They found a stronger SNARC-like effect in the putative subitizing range (1–4), compared to larger numerosities (6–9) for the orientation judgement only.

In a recent study, Zhou et al. [25] used a same/different matching task to investigate the spatial representation of non-symbolic magnitude, size and density, to determine if non-symbolic magnitude is spatially represented, as evidenced by a non-symbolic SNARC effect. In the non-symbolic magnitude and density matching task, two dot arrays were presented sequentially. The first dot array was presented for 200 ms, followed by a blank screen for 1300 ms, followed by the second dot array that remained until a response was made (arrays comprised 11, 14, 17, 20, 23, 26 and 29 dots). In the area matching task, stimuli comprised pentagons differing in size. Findings revealed faster right-hand responses to large non-symbolic magnitudes; however, no difference was found for left hand responses. The authors suggest this was due to the right-hand responses reducing the RT difference between small left and small right data. Neither size nor density affected responses. The authors claimed their findings are consistent with a non-symbolic SNARC effect.

While Zhou et al. [25] overcome potential interpretive limitations of earlier non-symbolic work by excluding quantities in the subitizing range, and by examining the potential influence of visual cues of area and density, the authors employed an independent group design in which each condition was completed by different participants. One potential issue is the design ignores possible individual differences in density and area judgements. Moreover, like other researchers, Zhou et al. collapsed data across different magnitudes (i.e. ‘small’ consisted of 11, 14 and 17 dots, while ‘large’ consisted of 23, 26 and 29 dots). Collapsing across data for non-symbolic stimuli may be problematic since there are known individual differences in magnitude processing abilities (see Chew, Forte & Reeve, [28]).

Some cognitive indices (e.g., working memory) are thought to affect the symbolic SNARC effect [29]. Differences in VSWM in children have been linked to poorer math accuracy [30] and the absence of a symbolic SNARC effect in those with comorbid VSWM disability and dyscalculia [31]. It is of interest whether there is a link between a non-symbolic SNARC effect and VSWM.

Not everyone exhibits a symbolic SNARC effect [32–38]. In symbolic SNARC research, data is commonly collapsed over ‘small’ and ‘large” arrays (i.e. 1, 2 and 3 might be deemed small and 7, 8, and 9 large), which may mask individual differences [32]. Moreover, we know little of factors associated with the presence/absence of a SNARC effect. Some studies have shown VSWM is related to non-symbolic magnitude processing [39] and other studies have found a relationship between math ability and symbolic SNARC effects [34, 36]; the relationship between non-symbolic number acuity and math ability is less consistent [40]. Given these findings, it is possible the presence/absence of a non-symbolic effect is related to VSWM and/or math abilities.

## The current study

To overcome possible methodological limitations in research that has attempted to identify a non-symbolic SNARC effect, and to determine whether non-symbolic SNARC effects are associated with visuo-spatial working memory and/or math ability, we employed a referent-to-target judgment paradigm, in which stimuli were in the ANS range. Participants were presented a referent dot array for 300 ms followed by a second dot array and judged whether the second array contained fewer or more dots than the referent array. The target judgment arrays (15, 20, 25, 36, 45 and 60 dots) were selected so the ratio between the smallest array (*n* = 15 dots) and the referent array (*n* = 30 dots) was the same as the ratio between the largest arrays (*n* = 60 dots) and the referent array. Similarly, the ratio between arrays containing 20 and 30 dots was the same as arrays containing between 30 and 45 dots, and the ratio between 25 and 30 dots was the same as between 30 and 36 dots. The rationale for the design is, all things being equal, difficulty discriminating two non-symbolic magnitudes reflects the ratio between them (discriminating 9 from 10 dots is as difficult as discriminating 90 from 100 dots; see Mazzocco, Feigenson & Halberda [41]).

The set sizes selected were based on ratios that putatively reflect similar levels of difficulty for pairs of stimuli (i.e., 15 and 60 dots = a ratio of 0.5, 20 and 45 dots = a ratio of 0.66, and 25 and 36 dots = a ratio of 0.83). Most research that has examined the symbolic SNARC effect has focused on distance effects. However, the meaning of “distance” using non-symbolic SNARC stimuli is less obvious. By focusing on ratio, we were able to investigate the effects of the relationship between ratio difficulties and non-symbolic SNARC effects. Like findings from magnitude discrimination studies using the Weber fraction measure, we expected the 0.5 ratio would be easiest to discriminate. We expected participants to respond more quickly to targets more distant from the referent array than targets closer to the referent array. Based on findings from symbolic SNARC research, we hypothesize smaller non-symbolic magnitudes would be associated with the left side of space and larger non-symbolic magnitudes with the right side of space, which, in turn, would be reflected in faster responses made with the left hand for smaller numerical magnitudes and for larger numerical magnitudes using the right hand. We also expected a hand by magnitude interaction. As working hypothesis, we expected the absence of a non-symbolic SNARC effect would be associated with poor VSWM and/or math ability. We used stimuli in the ANS range to avoid the use of sub-vocal strategies.

## Method

### Participants

Twenty-eight undergraduate psychology students participated for course credit. Participants (10 males, 18 females) were between 17 and 36 years (*M* = 20.32 years, *SD* = 3.9) and, as measured by the Edinburgh Handedness Inventory questionnaire (Oldfield), were strongly right handed. While the sample comprised students from a range of cultural backgrounds, all spoke English fluently. All participants had normal or corrected-to-normal vision. The study was conducted in compliance with the approval and requirements of the author’s University’s Human Ethics Committee, University of Melbourne (# 1237507.2). All participants provided written, informed consent.

### Materials and procedure

All participants completed a non-symbolic magnitude judgement, Corsi Blocks Forwards (VSWM), WRAT-4 Math Computational Subtest (Mathematics ability), and the Edinburgh Handedness Inventory (Handedness) tasks. The non-symbolic magnitude judgement task was presented on a 20” computer screen using E-Prime software (version 2.0), with a viewing distance of approximately 50cm at ~ 8 degrees’ visual angle. Dot arrays were generated using Microsoft Paint software.

#### Corsi Blocks-VSWM

The Corsi Blocks VSWM test was administered following Kessels, van Zandvoort, Postma, Kappelle, and de Haan’s [42] method. The examiner began by a tapping a two-block sequence. The participant tapped the same sequence back. Sequences gradually increased in the number of blocks tapped and task ended when the participant failed to correctly copy a sequence twice in a row. Block span was calculated as the longest block sequence repeated correctly.

#### Wide Range Achievement Test -4th Edition (WRAT-4)—math ability

*WRAT-4*, *Math Computational Subtest*–(Wilkinson & Robinson, [43]) comprises 40-items and assesses the capacity for basic mathematic skills though counting, identifying numbers, carrying out basic written math problems such as addition, subtraction, division, multiplication, fractions, decimals and algebra.

#### Edinburgh Handedness Inventory (Oldfield, [44])—Handedness

This 10-item questionnaire indexes hand preference. Responses range from strong (++) through to less strong (+) and indifferent (+/-).

#### Non-symbolic magnitude judgement task

**Fig 1:**
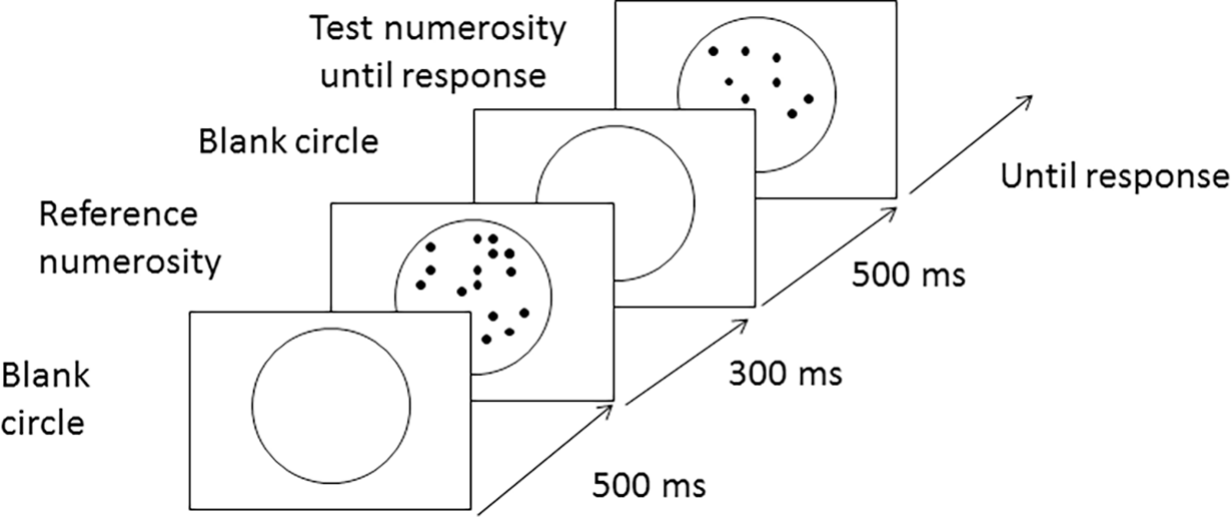
Diagram of procedure.

Firstly, a blank screen was shown, followed by a grey circle in the centre of the screen for 500 ms. On each trial, participants were presented with two dot displays in sequence (first the referent stimulus for 300ms, followed by the target until response) (see Fig 1 below for the trials sequence). These presentation times were chosen after pilot work showed participants could not enumerate the dots in this time. That is response times indicated they were not enumerating. The first dot display (the referent stimulus) always contained a fixed number of dots (black dots). The second dot display (the target stimulus) contained variable numbers of dots (15, 20, 25, 36, 45, or 60 dots). Participants judge whether the second arrays of dots (the target stimulus) contained *fewer* or *more* dots than the referent.

Participants responded by pressing either a left or a right response button, as appropriate. Each participant completed two blocks of 96 trials (192 trials in total). Hand of response was counterbalanced. In Block 1, participants were asked to press the left button if the target stimulus contained *less* dots and the right button if the target contained *more* dots. For Block 2, the instructions for the button response were reversed so that a left button press indicated a *more* response for the target stimulus and a right button press indicated a *less* response for the target stimulus. Participants were asked to switch their hand of response for the second half of the experiment.

Prior to each block of trials, participants completed 20 practice trials. In the practice session, accuracy was shown as a percentage on the screen. Participants with an accuracy of less than 75 percent were required to conduct the practice session again. Participants were given a short break at the halfway point, prior to switching response hand. After the magnitude judgment task, participants completed the WRAT math and VSWM tasks.

## Analytic approach

Data analysis in symbolic SNARC studies commonly use ANOVA [2, 6, 45, 46] or linear regression [47] techniques. In ANOVAs a SNARC effect is defined as the interaction between hand and magnitude [1]. The SNARC effect is not contingent on the hand used during motor response selection (Dehaene et al., Exp. 6, [1]), is independent of effector used [48, 49], and appears to be amodal [6]. The SNARC effect has also been defined as the difference between congruent and incongruent response conditions and spatial mapping [50–52]. While ANOVAs test if a SNARC effect is present, and is useful for group-level analysis, it does not identify individual differences in RT latencies or accuracy. One way of overcoming this issue is to examine difference in right-hand RTs and left-hand RTs in a linear regression, in which a SNARC effect is defined as a negative slope.

To test for the presence of a non-symbolic SNARC-like interaction effect, we conducted a 2 × Response Key Position (left or right-side response box) × 2 Direction of the Magnitude/Referent Relationship (smaller or larger than the 30-dot referent array) × 3 Ratios (0.5, 0.66, 0.83) ANOVA with Bonferroni post-hoc contrasts. (It should be noted that we assessed the data distribution characteristics and found the RT distributions to be normal in form, as a consequence of which we did not transform the data, and the findings reported herein are based on the untransformed RT data.) We also examined the influence of the three different ratios (0.5, 0.66 and 0.83) on the non-symbolic SNARC-like effect in separate 2 × Response Key Position (left or right-side response box) × 2 Direction of the Magnitude/Referent Relationship (smaller or larger than the 30-dot referent array) ANOVAs. Incorrect trials were excluded from analysis, as were trials where response times (RTs) of 200ms or less (often considered to be anticipatory responses) and trials which exceeded 1000ms (1000ms was chosen since it was more than 3SDs above the mean RT).

For each participant, twelve median reaction times (RTs) were derived for left and right-hand responses to each of the target stimuli (15, 20, 25, 36, 45 and 60 dots). There were four experimental conditions: (1) left-hand response to target stimuli with smaller non-symbolic magnitude (e.g., a referent containing 30 dots and a target containing 15, 20 or 25 dots); (2) left-hand response to target stimuli with larger non-symbolic magnitude (e.g. a reference containing 30 dots and a target containing 36, 45, or 60 dots; and (3) right-hand response to target stimuli with smaller non-symbolic magnitude; and (4) right-hand response to target stimuli with larger non-symbolic magnitude. Three median RTs from the four experimental conditions were analysed using an ANOVA.

To examine individual differences in the direction of a non-symbolic SNARC-like effect we followed Fias, Brysbaert, Geypens, and d,Ydewalle ‘s [47] analytic method. The difference between right and left RTs (dRTs) were entered into a linear regression analysis in which the natural logarithm of the non-symbolic magnitude was the independent variable. The linear equation y = ax + b was used to calculate the slope for each individual. β slope coefficients were analysed using a t-test to determine whether slopes were significantly different from 0.

A negative slope indicates a standard SNARC effect (i.e., small non-symbolic magnitudes associated with the left-hand side), and a positive slope would indicate a reverse SNARC effect (small non-symbolic magnitudes associated with the right-hand side).

We also examined the relationship between VSWM, math ability and the non-symbolic SNARC-effect to determine whether these factors were associated with performance on the non-symbolic SNARC task.

## Results

The median RTs and accuracy for Magnitude × Hand judgments are shown in Table 1. Table 1 reveals that, as the targets get closer in magnitude to the referent, responses appear to slow and are less accurate.

**Table 1:** Medians and SE’s of RTs and means and SD’s of accuracy as a function of magnitude.

			Left Hand				Right Hand			
Ratio	Dot Magnitude	Target to Referent	RTs (ms)		Accuracy		RTs (ms)		Accuracy	
			*Mdn*	*SE*	*M*	*SD*	*Mdn*	*SE*	*M*	*SD*
0.5	15	0.5 x Ref	497.50	17.49	0.99	0.03	518.75	17.76	0.99	0.02
0.66	20	1/1.5 x Ref	578.00	17.71	0.95	0.07	559.00	18.20	0.97	0.04
0.83	25	1/1.2 x Ref	641.25	21.85	0.80	0.20	664.50	20.57	0.78	0.20
0.83	36	1.2 x Ref	555.75	23.08	0.93	0.09	522.75	18.24	0.92	0.11
0.66	45	1.5 x Ref	521.25	21.04	0.97	0.05	461.25	17.13	0.97	0.04
0.5	60	2 x Ref	494.00	51.04	1.00	0.01	442.25	15.67	0.99	0.02

### SNARC effect for non-symbolic numerical magnitudes

Initial analysis showed no error rate interaction between magnitude and hand (*F* (1, 27) = .95, *p* = .34, ƞ^2^ = .03) (i.e., no evidence of a speed-accuracy trade off: the mean error rate was 7.37%). We conducted a repeated-measures ANOVA to determine whether a SNARC-like effect was evident for non-symbolic numerical magnitudes. In this analysis we used the Greenhouse-Geisser correction of the data since Mauchley’s test was significant χ^2^ (2) = 15.00, *p* < .05, which suggests the assumption of sphericity had been violated. The analysis comprised a 2 × Response Key Position (left or right-side response box) × 2 Direction of the Magnitude/Referent Relationship (smaller or larger than the 30-dot referent array) × 3 Ratio (0.5, 0.66, 0.83) ANOVA. The analysis revealed that Hand was not significant (*F*(1, 27) = 3.15, *p* = .09). However, Magnitude was significant (*F*(1,27) = 89.31, *p* < .0001, ƞ^2^ = .77) as was Ratio (*F*(1.39, 37.54) = 94.25, *p* < .0001, ƞ^2^ = .78). This finding suggested there was a difference in responses for small and large magnitudes and for differing ratios. We also found a significant interaction between Hand and Magnitude (*F*(1,27) = 6.29, *p* = .018, ƞ^2^ = .19), which supports the existence of a non-symbolic SNARC-like effect. The Magnitude × Ratio interaction was also significant (*F*(1.60, 43.05) = 17.20, *p* < .0001, ƞ^2^ = .39). However, neither the Hand × Ratio (*F*(1.65, 44.54) = 1.58, *p* = .22) nor the three-way interaction for Hand × Magnitude × Ratio were significant (*F*(1.86, 50.17) = .48, *p* = .61).

Since the main effect for ratio was significant, we compared differences in accuracy and RT for the three ratios. The analysis showed that targets further in magnitude from the referent were judged more quickly and more accurately. Bonferroni post-hoc contrasts revealed that targets furthest in magnitude from the referent (a ratio of 0.5) were responded to significantly faster (36 ms faster) than the next ratio closer to the referent (a ratio of 0.66) and 102 ms faster than the ratio closest to the reference (a ratio of 0.83). (all contrasts *p* < .05). This suggests that examining the three ratios separately would be more informative about the nature of the non-symbolic SNARC effect.

### Ratio and the SNARC Effect

Three separate 2 x 2 ANOVAs were conducted to assess the relationship between response hand (left hand vs right hand) and non-symbolic magnitude RTs for the three ratios (0.5, 0.66 and 0.83 respectively).

The analysis of the 0.5 ratio showed a Magnitude effect (*F*(1, 27) = 40.26, *p* = < .0001, ƞ^2^ = .60; and 60 dots were responded to faster than 15 dots. While no effects was found for Hand (*F*(1, 27) = 1.85, *p* = . 19), the interaction between Magnitude and Hand was significant (*F*(1, 27) = 6.141 *p* = .010, ƞ^2^ = .19). This provided support for a non-symbolic SNARC-like effect for the 0.5 ratio. Simple effects analysis showed left hand responses showed no difference for small or large numerosities (*F*(1,27) = 1.46, *p* = 0.24), whereas participants responded faster using their right hand (a 38 ms advantage for right hand responses) to larger compared to smaller numerosities (*F*(1,27) = 9.52, *p* = 0.01, ƞ^2^ = 0.26).

The analysis of the 0.66 ratio showed a Magnitude effect (*F*(1, 27) = 49.44, *p* < .0001, ƞ^2^ = .65), and a Hand effect (*F*(1, 27) = 8.58, *p* = .01, ƞ^2^ = .24 –the right hand was faster than the left hand), and an interaction between Magnitude and Hand (*F*(1, 27) = 7.84, *p* = .01, ƞ^2^ = .23). Although, for the 0.66 ratio, the left hand was not faster than the right for smaller numerosities.

The 0.83 ratio analysis showed a Magnitude effect (*F*(1, 27) = 56.54, *p* < .0001, ƞ^2^ = .68), no effect of Hand (*F*(1, 27) = .12, *p* = . 73), but a marginally significant interaction between Magnitude and Hand (*F*(1, 27) = .2.96, *p* = .10 (two-tailed) or .05 (one-tailed), ƞ^2^ = .10).

Following the analytic convention used in symbolic SNARC studies, we collapsed data across small (15, 20, 25) and large (36, 45, 60) magnitudes. Results still showed support for a SNARC-like effect. Hand was not significant (*F*(1, 27) = 3.17, *p* = .09). However, Magnitude (*F*(1,27) = 89.12, *p* < .0001, ƞ^2^ = .77) and the Hand × Magnitude Interaction were significant (*F*(1,27) = 6.30 *p* = .02, ƞ^2^ = .19).

**Fig 2:**
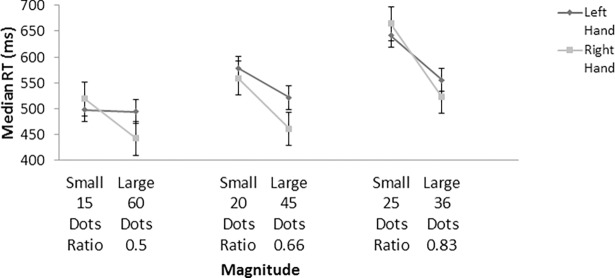
Median RTs comparing 15 vs 60, 20 vs 45 and 25 vs 36 dots to the 30 dot referent as a function of left vs right hand (error bars = standard error).

See Fig 2 below for the Hand by Magnitude interaction effect for the three separate ratios (0.5, 0.66 and 0.83). Fig 2 shows the form of the non-symbolic SNARC-like effect differs for each ratio but is similar to the symbolic SNARC effect for the 0.5 and 0.66 ratios.

### SNARC, VSWM and Math Abilities

The WRAT standardised score ranged from 87 to 143 with a mean of 113.14. This showed for the sample above average mathematics ability according to WRAT norms. For the Corsi Blocks task, visuospatial span ranged from 5 to 9 with a mean of 6.54. The average test norm for Corsi span is 6. Correlation coefficients for the WRAT, Corsi span, and regression slope are reported in Table 2 below. Table 2 shows that there were no significant correlations with either VSWM (Corsi span task) or math ability (WRAT) and the reaction time task (slope).

**Table 2:** Correlations between VSWM, math measures and β slope.

	WRAT	Corsi Span	β Slope
WRAT	-		
Corsi Span	0.09	-	
β Slope	0.05	0.07	-

Note: *N* = 28, WRAT = Wide Range Achievement Test

To investigate the relationship between VSWM and math ability and the non-symbolic SNARC-like effect, we conducted a 2 × Response Key Position (left or right-side response box) × 2 Direction of the Magnitude/Referent Relationship (smaller or larger than the 30-dot referent array) × 3 Ratio (0.5, 0.66, and 0.83) repeated measures ANCOVA. The math measure (WRAT standardised score) and VSWM measures (Corsi) were entered as covariates. The interaction effect between Hand and Magnitude was significant (*F*(1,27) = 8.08, p = . 01, ƞ^2^ = . 23) suggesting math and VSWM were not significant predictors of the non-symbolic SNARC effect. As the earlier analysis showed that only the 0.5 ratio showed a non-symbolic SNARC effect, we ran a 2 x Response Key Position (left or right-side response box) × 2 Direction of the Magnitude/Referent Relationship (smaller or larger than the 30-dot referent array) repeated measures ANCOVA for this ratio. The math (WRAT standardised score) and VSWM measures (Corsi) were entered as covariates. The original interaction effect for Hand by Magnitude remained significant (*F*(1,27) = 6.14, p = .020, ƞ^2^ = .19). This suggested that for the 0.5 ratio both math and VSWM measures were not significant predictors of the non-symbolic SNARC effect.

### Standard and reverse SNARC effects

Inspection of participants’ performances showed that a small number of individuals (*n* = 8) exhibited a reverse non-symbolic SNARC effect, with an average regression slope β value of 66.44, indicating a positive slope which significantly differed from 0, (*t*(7) = 4.17, *p* = .004). Participants who exhibited a standard SNARC effect (*n* = 19), had an average regression slope β value of -114.33. This indicated a negative slope which significantly differed from 0, (*t*(18) = 5.39, *p* < .0001). One participant had a regression slope of .00, indicating a flat line and no SNARC effect.

An independent samples t-test confirmed a large difference in β’s -180.77 ms for those individuals who exhibited a standard SNARC compared to a reverse SNARC effect. This difference between subgroups was significant (*t*(24.22) = 6.81, *p* < .0001).

### Standard SNARC/Reverse SNARC, VSWM and math ability

We compared the VSWM and WRAT math measures for individuals who exhibited a standard or a reverse SNARC effect. These analyses revealed no differences in either VSWM or math between the two groups. For the math task, an independent samples t-test showed participants in the standard SNARC subgroup (*M* = 114.89, *SD* = 12.73) scored 5.52 points higher on the WRAT on average than those in the reverse SNARC subgroup (*M* = 109.38, *SD* = 18.72). However, this difference was not significant (*t*(25) = .89, *p* = .38). For the VSWM task, an independent samples t-test showed little difference (*t*(25) = .06, *p* = .96) between participants in the standard SNARC subgroup (*M* = 6.47, *SD* = 1.22) and those in the reverse SNARC subgroup (*M* = 6.5, *SD* = 0.93).

## Discussion

This study investigated whether a non-symbolic SNARC effect is evident in large magnitude dot arrays in a referent-to-target judgement paradigm. Three findings are worth noting. First, findings show non-symbolic magnitude is represented spatially, as evidenced by a non-symbolic SNARC effect. Presentation of smaller non-symbolic magnitudes (i.e., 15, 20, and 25) facilitated faster responses using the left hand, and larger non-symbolic magnitudes (i.e., 36, 45 and 60) facilitated faster responses using the right hand, demonstrating non-symbolic magnitude RTs is modulated by response hand. This finding confirmed those of Zhou et al. [25] showing a non-symbolic SNARC effect. In other words, a non-symbolic SNARC effect is evident in the so-called ANS range. A distance effect was also evident: participants were quicker responding to targets further away from the referent dot arrays. For dot arrays closer in magnitude to the referent, responses slowed.

Second, the form of the interaction in the non-symbolic SNARC effect showed a similar pattern to the symbolic SNARC effect (i.e., an interaction effect for non-symbolic magnitude and hand). The difference between left-hand and right-hand RT responses to larger stimuli was more pronounced than left and right-hand responses to smaller stimuli.

Third, only 64% of participants showed a SNARC effect. This finding is similar to those found in symbolic SNARC research, which show just over half of participants exhibited a SNARC effect [32–38]. Indeed, atypical differences in ANS processing abilities more generally are common [53–55].

Given the pattern of findings is similar to symbolic SNARC studies, what factor distinguishes individuals who exhibit a SNARC effect from those who do not? Does the failure to exhibit a SNARC effect, for example, indicate a deficit in number space representation that, in turn, affects numerical processing abilities? There are three aspects to this question (participants assessed herein showed variation in RTs and SNARC slope direction—see the Supplementary Materials section): to what degree are differences in SNARC abilities related to differences in (1) number-space representation, (2) stimuli parameters, and (3) cognitive and/or number specific abilities. Answers to these questions may help clarify understanding of number-space relationships and the functional value of SNARC data more specifically.

The issue of how best to conceptualize number-space relationships has been raised by Leibovich, Katzin, Harel and Henik [56] who claim a sense of magnitude is the basis of math understanding. Specifically, they argue continuous magnitudes, such as size, area and density, are processed quicker and more accurately than number itself. In fact, they argue it is impossible to separate numerosity from continuous magnitudes in non-symbolic magnitude judgment tasks. Leibovich et al.’s claims are disputed, however. Burr [57], for example, provides evidence showing number strongly influences both area and density, and suggests number is a more fundamental attribute than continuous magnitudes. Similarly, Beran and Parrish [58] argued that although numerical and continuous magnitudes co-vary, continuous magnitudes alone do not influence responses. Indeed, Zhou et al., [25] showed a non-symbolic SNARC effect for non-symbolic numerical magnitudes, but not for continuous magnitudes (i.e., for area and density). These findings suggest it is unlikely a sense of magnitude alone is responsible for differences in non-symbolic SNARC abilities.

ANS theory (Dehaene, [59]) suggests the magnitude of numerosities is extracted independent of visual cues. In contrast, sensory integration theory (Gebuis, Kadosh & Gevers, [60]) proposes visual cues may affect magnitude judgment processes. Indeed, Leibovich et al., [56] argue that magnitude processing is affected by visual cues such as density, luminance and surface area. Since we did not control for low level visual cues, we are unable to draw conclusions about the role of these visual cues in the current study.

Individual differences in SNARC responses may be related to differences in how non-symbolic number is encoded and numerical decisions made. Odic [61] argues that non-symbolic numerical magnitude is encoded with the aid of low level congruent visual cues. For example, if density is manipulated it may influence perception of number because of shared visual cues; that is, numeric and non-numeric cues compete for decision components such as working memory. Odic’s arguments are similar to those of Gebuis and Reynvoet [62] who suggest visual cues may affect non-symbolic magnitude abilities. Gebuis et al., [60] argue for a sensory integration theory in which the importance of visual cues is weighted and these weights affect magnitude judgements [63]. Insofar as the impact of visual cues can be demonstrated, they might explain reasons for individual differences in non-symbolic SNARC judgments.

Insofar SNARC abilities are related to magnitude representations more generally, research has found links with magnitude representation, cognitive and math abilities. A study by Cornu, Hornung, Schiltz and Martin [64] discovered spatial skills partially mediated the relationship between number line estimation and arithmetic in kindergarteners. Also, a model with control variables (i.e. age, gender), domain-general skills (i.e. verbal short-term memory and verbal intelligence), domain-specific skills (i.e. counting knowledge, Arabic number knowledge and quantitative knowledge) and spatial skills (i.e. spatial perception, visuo-motor integration and spatial visualisation) was a better predictor of number line estimation and arithmetic four months later than a similar model without spatial skills [64]. Lourenco, Aulet, Ayzenberg, Cheung and Holmes [65], found individual differences in the associations between number, area and duration at 9 months of age predicted similar associations at 3.5 years. This finding shows stable individual differences in number magnitude representations over time. Arguably, non-symbolic numerical magnitude abilities represent a way of acquiring the meaning of Arabic numbers symbols.

A large-scale longitudinal study by Tosto et al., [66] examined individual differences in number sense, its relation to math performance, and other general cognitive measures such as VSWM (Corsi), RTs (speed and accuracy), non-verbal intelligence (Raven’s progressive matrices), vocabulary (Mill Hill Vocabulary) and reading comprehension. The aim of the study was to examine which measures were important, at what time during development, how strong the relationship was and to what degree these measures were moderated by other cognitive measures. To do this, Tosto et al. tested the estimation ability of 4,984 16-year-old students using number sense measures (i.e. a dot comparison and number line task). This data was compared to cognitive and achievement data from the same students at ages 7, 9, 10, 12 and 14. Results of the study showed dot comparison and number line tasks correlated with mathematics performance at age 16. However, the strength of the association between dot comparison, number line tasks and earlier math performance varied considerably across development and was moderated by differing cognitive abilities [66]. This shows both domain-general (quantitative) and domain-specific (working memory, spatial) skills are related to magnitude representation and development of math.

Individual differences in the symbolic SNARC effect and number-space mapping may be related to structural differences in the brain. For example, Krause, Lindemann, Toni and Bekkering [67] showed participants with a stronger SNARC effect had increased grey matter in the parietal area of the right precuneus. Amalric and Dehaene [68] showed bilateral activation of brain areas related to numbers and space, but little activation of language areas in expert mathematicians carrying out high level math reasoning. Amalric and Dehaene suggest this finding supports the claim that number-space mapping in early development is related to math achievement. A recent literature review by Myers, Carey and Szucs [69] on the neural correlates of spatial processing and WM in gifted children and adults showed these abilities contributed to being gifted at math.

Indeed, adults with developmental dyscalculia have shown deficits mapping number onto physical space [70]. Some children with visuo-spatial deficits and developmental dyscalculia show a reversed symbolic SNARC effect (suggesting a right-to-left mental number-line) and poorer math performance, compared to matched controls who show a standard SNARC effect [31].

Training in spatial associations improves acuity and math performance [71]. As far as we can ascertain, no studies have investigated the impact of magnitude representation training on changes in SNARC abilities. To more thoroughly investigate the significance of individual differences in SNARC and associated abilities, different analytic methods may be helpful. For example, Chew, Forte and Reeve [28] conducted a latent class analysis of symbolic and non-symbolic magnitude judgments, which revealed distinct and different patterns of judgments associated with math abilities.

## Conclusion

The findings confirm a non-symbolic SNARC effect which, in turn, supports the claim that non-symbolic magnitude information is represented mentally in a left-to-right spatial manner. While the focus of the present study was non-symbolic representation, caution should be exercised in drawing parallels between factors that affect differences in non-symbolic and symbolic SNARC effects. It might be not only easier to differentiate 60 and 66 presented as Arabic symbols, compared to presented as dot arrays, but it seems likely different factors might affect the speed of judgments in the two representations [28]. Future researchers would do well to identify those factors that affect individual differences for both symbolic and non-symbolic SNARC judgments.

## Supporting information

S1 File Supplementary information. (DOCX)

## References

[1] Dehaene S, Bossini S, Giraux P. The mental representation of parity and number magnitude. Journal of Experimental Psychology: General. 1993;122(3):371–96. 10.1037/0096-3445.122.3.371

[2] Keus IM, Jenks KM, Schwarz W. Psychophysiological evidence that the SNARC effect has its functional locus in a response selection stage. Cognitive Brain Research. 2005;24(1):48–56. 10.1016/j.cogbrainres.2004.12.005 15922157

[3] Gevers W, Lammertyn J, Notebaert W, Verguts T, Fias W. Automatic response activation of implicit spatial information: Evidence from the SNARC effect. Acta Psychologica. 2006;122(3):221–33. 10.1016/j.actpsy.2005.11.004 16423320

[4] Gevers W, Santens S, Dhooge E, Chen Q, Van den Bossche L, Fias W, et al Verbal-spatial and visuospatial coding of number–space interactions. Journal of Experimental Psychology: General. 2010;139(1):180. 20121318 10.1037/a0017688

[5] Fattorini E, Pinto M, Rotondaro F, Doricchi F. Perceiving numbers does not cause automatic shifts of spatial attention. Cortex. 2015;73:298–316. 10.1016/j.cortex.2015.09.007 26520681

[6] Nuerk HC, Wood G, Willmes K. The universal SNARC effect—The association between number magnitude and space is amodal. Experimental Psychology. 2005;52(3):187–94. 10.1027/1618-3169.52.3.187 16076066

[7] Reynvoet B, Brysbaert M. Single-digit and two-digit Arabic numerals address the same semantic number line. Cognition. 1999;72(2):191–201. 10.1016/S0010-0277(99)00048-7 10553671

[8] Shaki S, Petrusic WM. On the mental representation of negative numbers: Context-dependent SNARC effects with comparative judgments. Psychon Bull Rev. 2005;12(5):931–7. 10.3758/BF03196788 16524013

[9] Shaki S, Gevers W. Cultural Characteristics Dissociate Magnitude and Ordinal Information Processing. Journal of Cross-Cultural Psychology. 2011;42(4):639–50. 10.1177/0022022111406100

[10] Ren P, Nicholls ME, Ma YY, Chen L. Size matters: non-numerical magnitude affects the spatial coding of response. PLoS One. 2011 8 11;6(8):e23553 10.1371/journal.pone.0023553 21853151 PMC3154948

[11] Shaki S, Petrusic WM, Leth-Steensen C. SNARC effects with numerical and non-numerical symbolic comparative judgments: Instructional and cultural dependencies. Journal of Experimental Psychology: Human Perception and Performance. 2012;38(2):515–30. 10.1037/a0026729 22288694

[12] Rusconi E, Kwan B, Giordano BL, Umiltà C, Butterworth B. Spatial representation of pitch height: the SMARC effect. Cognition. 2006;99(2):113–29. 10.1016/j.cognition.2005.01.004 15925355

[13] Nishimura A, Yokosawa K. Effects of laterality and pitch height of an auditory accessory stimulus on horizontal response selection: The Simon effect and the SMARC effect. Psychon Bull Rev. 2009;16(4):666–70. 10.3758/PBR.16.4.666 19648450

[14] Vicario C, Rumiati R. Left-right compatibility in the processing of trading verbs. Frontiers in Behavioral Neuroscience. 2014;8(16). 10.3389/fnbeh.2014.00016 24478662 PMC3904129

[15] Holmes KJ, Lourenco SF. Common spatial organization of number and emotional expression: A mental magnitude line. Brain and Cognition. 2011;77(2):315–23. 10.1016/j.bandc.2011.07.002 21839568

[16] Macnamara A, Keage HAD, Loetscher T. Mapping of non-numerical domains on space: a systematic review and meta-analysis. Exp Brain Res. 2018;236(2):335–46. 10.1007/s00221-017-5154-6 29279982

[17] Dehaene S, Piazza M, Pinel P, Cohen L. Three parietal circuits for number processing. Cognitive Neuropsychology. 2003;20(3–6):487–506. 10.1080/02643290244000239 20957581

[18] Harvey BM, Klein BP, Petridou N, Dumoulin SO. Topographic Representation of Numerosity in the Human Parietal Cortex. Science. 2013;341(6150):1123–6. 10.1126/science.1239052 24009396

[19] Bueti D, Walsh V. The parietal cortex and the representation of time, space, number and other magnitudes. Philosophical Transactions of the Royal Society B: Biological Sciences. 2009;364(1525):1831–40. 10.1098/rstb.2009.0028 19487186 PMC2685826

[20] Dehaene S, Brannon E, editors. Space, time and number in the brain: Searching for the foundations of mathematical thought London: Academic Press; 2011.

[21] Hubbard EM, Piazza M, Pinel P, Dehaene S. Interactions between number and space in parietal cortex. Nature Reviews Neuroscience. 2005;6:435 10.1038/nrn1684 15928716

[22] Walsh V. A theory of magnitude: common cortical metrics of time, space and quantity. Trends in Cognitive Sciences. 2003;7(11):483–8. 10.1016/j.tics.2003.09.002 14585444

[23] Mitchell T, Bull R, Cleland AA. Implicit response-irrelevant number information triggers the SNARC effect: Evidence using a neural overlap paradigm. The Quarterly Journal of Experimental Psychology. 2012;65(10):1945–61. 10.1080/17470218.2012.673631 22524699

[24] Patro K, Haman M. The spatial–numerical congruity effect in preschoolers. Journal of Experimental Child Psychology. 2012;111(3):534–42. 10.1016/j.jecp.2011.09.006 22153910

[25] Zhou X, Shen C, Li L, Li D, Cui J. Mental Numerosity Line in the Human’s Approximate Number System. Experimental psychology. 2016 10.1027/1618-3169/a000324 27404985

[26] Hyde DC, Spelke ES. Neural signatures of number processing in human infants: evidence for two core systems underlying numerical cognition. Developmental science. 2011;14(2):360–71. 10.1111/j.1467-7687.2010.00987.x 21399717 PMC3050652

[27] Feigenson L, Dehaene S, Spelke E. Core systems of number. Trends in Cognitive Sciences. 2004;8(7):307–14. 10.1016/j.tics.2004.05.002 15242690

[28] Chew CS, Forte JD, Reeve RA. Cognitive factors affecting children’s nonsymbolic and symbolic magnitude judgment abilities: A latent profile analysis. Journal of Experimental Child Psychology. 2016;152:173–91. 10.1016/j.jecp.2016.07.001 27560661

[29] van Dijck J-P, Fias W. A working memory account for spatial–numerical associations. Cognition. 2011;119(1):114–9. 10.1016/j.cognition.2010.12.013 21262509

[30] Crollen V, Noël M-P. Spatial and numerical processing in children with high and low visuospatial abilities. Journal of Experimental Child Psychology. 2015;132:84–98. 10.1016/j.jecp.2014.12.006 25618380

[31] Bachot J, Gevers W., Fias W., & Roeyers H. Number sense in children with visuospatial disabilities: Orientation of the mental number line. Psychology science. 2005;47(1):172. doi: 1854/LU-326765.

[32] Beecham R, Reeve RA, Wilson SJ. Spatial Representations Are Specific to Different Domains of Knowledge. PLoS ONE. 2009;4(5):e5543 10.1371/journal.pone.0005543 19461994 PMC2678257

[33] Cipora K, Nuerk H-C. Is the SNARC effect related to the level of mathematics? No systematic relationship observed despite more power, more repetitions, and more direct assessment of arithmetic skill. The Quarterly Journal of Experimental Psychology. 2013;66(10):1974–91. 10.1080/17470218.2013.772215 23473520

[34] Cipora K, Hohol M, Nuerk H-C, Willmes K, Brożek B, Kucharzyk B, et al Professional mathematicians differ from controls in their spatial-numerical associations. Psychological Research. 2016;80(4):710–26. 10.1007/s00426-015-0677-6 26063316 PMC4889706

[35] Fischer MH. The future for SNARC could be stark…. Cortex. 2006;42(8):1066–8. 10.1016/S0010-9452(08)70218-1 17209412

[36] Hoffmann D, Mussolin C, Martin R, Schiltz C, Lappe M. The Impact of Mathematical Proficiency on the Number-Space Association. PLoS One. 2014;9(1):e85048 10.1371/journal.pone.0085048 24416338 PMC3885673

[37] Viarouge A, Hubbard EM, McCandliss BD. The Cognitive Mechanisms of the SNARC Effect: An Individual Differences Approach. PLoS ONE. 2014;9(4):1–10. 10.1371/journal.pone.0095756 24760048 PMC3997411

[38] Wood G, Willmes K, Nuerk H-K, Fischer MH. On the cognitive link between space and number: A meta-analysis of the SNARC effect. Psychology science quarterly. 2008;50(4):489.

[39] Formoso J, Barreyro JP, Jacubovich S, Injoque-Ricle I. Possible Associations between Subitizing, Estimation and Visuospatial Working Memory (VSWM) in Children. The Spanish Journal of Psychology. 2017;20 Epub 06/05. 10.1017/sjp.2017.23 28578725

[40] Chen Q, Li J. Association between individual differences in non-symbolic number acuity and math performance: A meta-analysis. Acta Psychologica. 2014;148(0):163–72. 10.1016/j.actpsy.2014.01.016 24583622

[41] Mazzocco MM, Feigenson L, Halberda J. Impaired acuity of the approximate number system underlies mathematical learning disability (dyscalculia). Child development. 2011;82(4):1224–37. 10.1111/j.1467-8624.2011.01608.x 21679173 PMC4411632

[42] Kessels RP, van Zandvoort MJ, Postma A, Kappelle LJ, de Haan EH. The Corsi Block-Tapping Task: standardization and normative data. Applied Neuropsychology. 2000;7(4):252–8. 10.1207/S15324826AN0704_8 11296689

[43] Wilkinson GS, Robertson G. Wide Range Achievement Test 4 (WRAT4). Psychological Assessment Resources, Inc, Lutz, FL 2006.

[44] Oldfield RC. The assessment and analysis of handedness: the Edinburgh inventory. Neuropsychologia. 1971;9(1):97–113. 10.1016/0028-3932(71)90067-4 5146491

[45] Fischer MH, Rottmann J. Do negative numbers have a place on the mental number line. Psychology Science. 2005;47(1):22–32.

[46] Schwarz W, Muller D. Spatial associations in number-related tasks—A comparison of manual and pedal responses. Experimental Psychology. 2006;53(1):4–15. 10.1027/1618-3169.53.1.4 16610269

[47] Fias W, Brysbaert M, Geypens F, d'Ydewalle G. The Importance of Magnitude Information in Numerical Processing: Evidence from the SNARC Effect. Mathematical Cognition. 1996;2(1):95–110. 10.1080/135467996387552

[48] Fischer MH, Warlop N, Hill RL, Fias W. Oculomotor Bias Induced by Number Perception. Experimental Psychology. 2004;51(2):91–7. 10.1027/1618-3169.51.2.91 15114901

[49] Schwarz W, Keus IM. Moving the eyes along the mental number line: Comparing SNARC effects with saccadic and manual responses. Perception & Psychophysics. 2004;66(4):651–64. 10.3758/bf03194909. 15311664

[50] Fischer MH, Mills RA, Shaki S. How to cook a SNARC: Number placement in text rapidly changes spatial–numerical associations. Brain and Cognition. 2010;72(3):333–6. 10.1016/j.bandc.2009.10.010 19917517

[51] Gevers W, Gevers E, Ratinckx W, De Baene W, Fias. Further Evidence that the SNARC Effect is Processed Along a Dual-Route Architecture. Experimental psychology. 2006;53(1):58–68. 10.1027/1618-3169.53.1.58 16610273

[52] Gibson L, Maurer D. Development of SNARC and distance effects and their relation to mathematical and visuospatial abilities. Journal of experimental child psychology. 2016;150:301–13. 10.1016/j.jecp.2016.05.009 27376924

[53] Dowker A. Individual differences in numerical abilities in preschoolers. Developmental Science. 2008;11(5):650–4. 10.1111/j.1467-7687.2008.00713.x 18801119

[54] Gray SA, Reeve RA. Preschoolers' Dot Enumeration Abilities Are Markers of Their Arithmetic Competence. PLoS ONE. 2014;9(4):e94428 10.1371/journal.pone.0094428 24714052 PMC3979837

[55] Paul JM, Reeve RA. Relationship between single digit addition strategies and working memory reflects general reasoning sophistication. Learning and Instruction. 2016;42:113–22. 10.1016/j.learninstruc.2016.01.011

[56] Leibovich T, Katzin N, Harel M, Henik A. From “sense of number” to “sense of magnitude”: The role of continuous magnitudes in numerical cognition. Behavioral and Brain Sciences. 2017;40 10.1017/S0140525X16000960 27530053

[57] Burr DC. Evidence for a number sense. Behavioral and Brain Sciences. 2017;40 Epub 07/27. 10.1017/S0140525X16002077 29342625

[58] Beran MJ, Parrish AE. The number sense is neither last resort nor of primary import. Behavioral and Brain Sciences. 2017;40 Epub 07/27. 10.1017/S0140525X16002065 29342651

[59] Dehaene S. The number sense: How the mind creates mathematics. New York: Oxford University Press; 1997.

[60] Gebuis T, Kadosh RC, Gevers W. Sensory-integration system rather than approximate number system underlies numerosity processing: A critical review. Acta Psychologica. 2016;171:17–35. 10.1016/j.actpsy.2016.09.003 27640140

[61] Odic D. The contributions of non-numeric dimensions to number encoding, representations, and decision-making factors. Behavioral and Brain Sciences. 2017;40 Epub 07/27. 10.1017/S0140525X1600220X 29342632

[62] Gebuis T, Reynvoet B. Generating nonsymbolic number stimuli. Behavior Research Methods. 2011;43(4):981–6. 10.3758/s13428-011-0097-5 21512872

[63] Gebuis T, Cohen Kadosh R, Gevers W. Why try saving the ANS? An alternative proposal. Behavioral and Brain Sciences. 2017;40 Epub 07/27. 10.1017/S0140525X16002107 29342626

[64] Cornu V, Hornung C, Schiltz C, Martin R. Different aspects of spatial skills and their relation to early mathematics. Poster presented at: Workshop on Domain-General and Domain-Specific Foundations of Numerical and Arithmetic Processing; 2016 9 28–30, Tübingen, Germany. doi: 10993/28747

[65] Lourenco SF, Aulet LS, Ayzenberg V, Cheung C-N, Holmes KJ. Right idea, wrong magnitude system. Behavioral and Brain Sciences. 2017;40 Epub 07/27. 10.1017/S0140525X16002156 29342631

[66] Tosto MG, Petrill SA, Malykh S, Malki K, Haworth C, Mazzocco MM, et al Number sense and mathematics: Which, when and how? Developmental psychology. 2017;53(10):1924 10.1037/dev0000331 28758784 PMC5611774

[67] Krause F, Lindemann O, Toni I, Bekkering H. Different Brains Process Numbers Differently: Structural Bases of Individual Differences in Spatial and Nonspatial Number Representations. J Cogn Neurosci. 2014;26(4):768–76. 10.1162/jocn_a_00518 24188366

[68] Amalric M, Dehaene S. Origins of the brain networks for advanced mathematics in expert mathematicians. Proceedings of the National Academy of Sciences. 2016;113(18):4909–17. 10.1073/pnas.1603205113 27071124 PMC4983814

[69] Myers T, Carey E, Szűcs D. Cognitive and Neural Correlates of Mathematical Giftedness in Adults and Children: A Review. Frontiers in Psychology. 2017;8(1646). 10.3389/fpsyg.2017.01646 29118725 PMC5661150

[70] Huber S, Sury D, Moeller K, Rubinsten O, Nuerk H-C. A general number-to-space mapping deficit in developmental dyscalculia. Research in developmental disabilities. 2015;43:32–42. 10.1016/j.ridd.2015.06.003 26151441

[71] Sella F, Tressoldi P, Lucangeli D, Zorzi M. Training numerical skills with the adaptive videogame “The Number Race”: A randomized controlled trial on preschoolers. Trends in Neuroscience and Education. 2016;5(1):20–9. 10.1016/j.tine.2016.02.002

