# Supplementary material for: Non-symbolic magnitudes are represented spatially: Evidence from a non-symbolic SNARC task
Source: PLoS One. 2018 Aug 30;13(8):e0203019. doi: 10.1371/journal.pone.0203019 (PMC6116986; doi:10.1371/journal.pone.0203019)
Supplement: S1 File — (DOCX) [file pone.0203019.s001.docx]

**Supplementary Information**

**Individual participant dRTs** The regression slope for each participant was examined separately. Figure 3 below shows dRTs plotted for each participant who met the 50% correct accuracy threshold for any given non-symbolic magnitude. Large individual differences can be seen by the slope of the regression lines. Some participants’ regression lines slope downward indicating a negative slope value and a standard SNARC-like effect or upward indicating a positive slope value and a reversed SNARC. A flat line with a slope value of 0 indicates no SNARC.


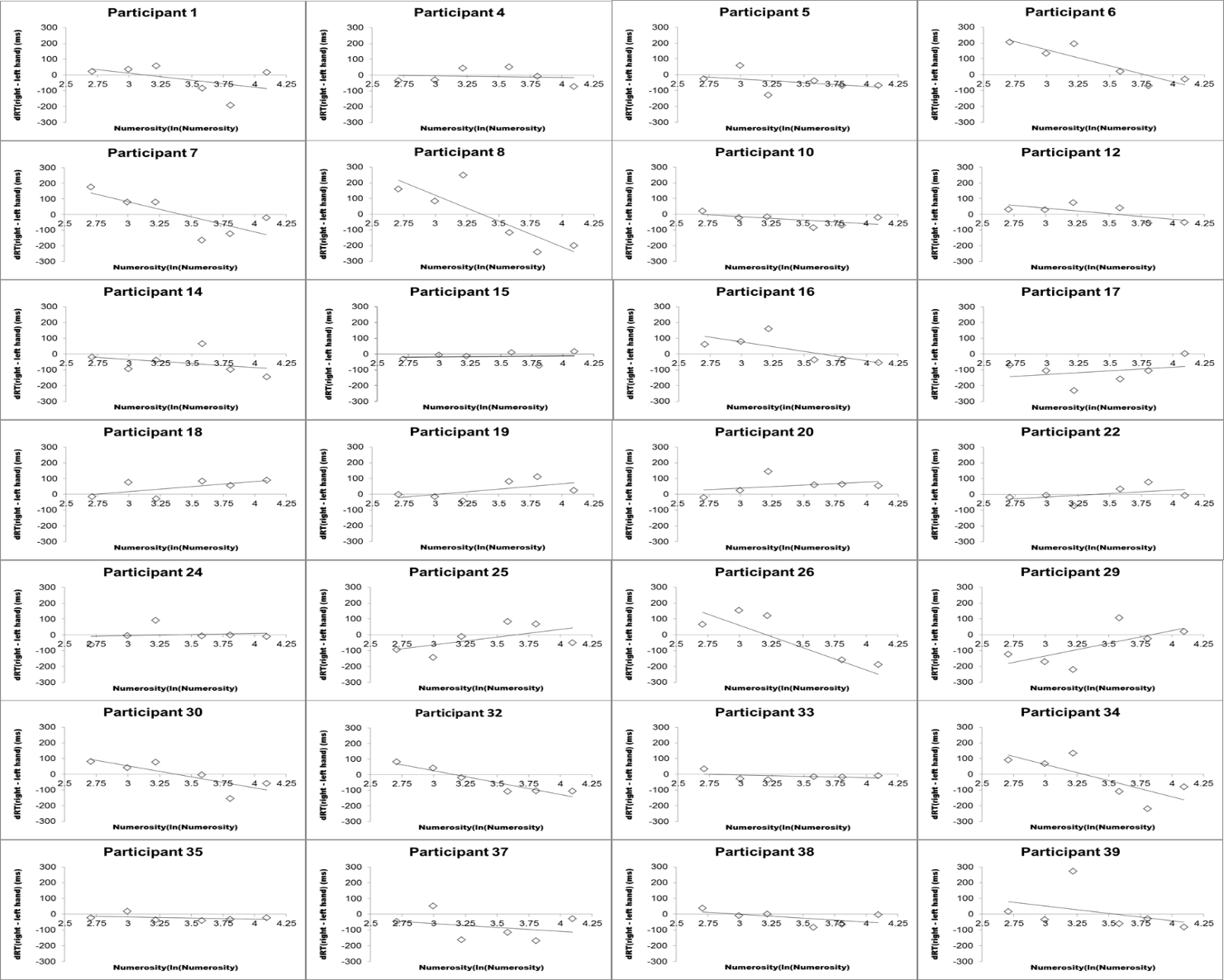


**Fig 3.** Individual differences of the non-symbolic SNARC-effect for participants depicting observed data and the regression line, reflecting response time differences for right and left hand as a function of non-symbolic magnitude. The x-axis value for each dot point represents the natural logarithm of the non-symbolic magnitude (i.e. 2.70 = ln(15), 3.00= ln(20), 3.22 = ln(25), 3.58 = ln(36), 3.81= ln(45), 4.09 = ln(60). When the gradient of the regression line slopes downward this indicates a SNARC effect. When the gradient of the regression line slopes upwards this indicates a reversed SNARC effect.
